# Identification and Pathogenicity Analysis of Feline Calicivirus in Shanghai and Guangdong, China

**DOI:** 10.1155/tbed/8729295

**Published:** 2025-06-04

**Authors:** Dan Luo, Weile Xie, Na Li, XueYan Peng, Kai Li, XinChu Zhou, Zhe Wang

**Affiliations:** ^1^Shanghai Key Laboratory of Veterinary Biotechnology, School of Agriculture and Biology, Shanghai Jiao Tong University, Shanghai, China; ^2^Collaborative Innovation Center of Agri-Seeds, School of Agriculture and Biology, Shanghai Jiao Tong University, Shanghai, China; ^3^Shanghai Veterinary Research Institute, Chinese Academy of Agricultural Sciences, Shanghai, China; ^4^Shanghai Shenpu Pet Hospital, Shanghai, China; ^5^Institute of Urban Agriculture, Chinese Academy of Agricultural Sciences, Chengdu, China

**Keywords:** broad-spectrum neutralization, challenge experiments, enteritis, feline calicivirus, phylogenetic analysis, VP1

## Abstract

Feline calicivirus (FCV; *Caliciviridae*) is a highly contagious RNA virus that causes upper respiratory tract infections and intestinal symptoms in cats. In 2023 and 2024, in Shanghai (SH), China, we collected oral swab samples from 189 domestic cats exhibiting symptoms of upper respiratory tract disease (URTD), to test for five designated respiratory pathogens. Among the 111 cats testing positive for these pathogens, the FCV-positivity rate was 52% (58/111). Six FCV strains (three from SH and three from Guangdong [GD]) were successfully isolated from respiratory specimens from domestic cats. Whole genome sequencing and phylogenetic analyses revealed that these strains exhibited the GII FCV genotype, suggesting that this is the dominant genotype in Asia. Within the first 36 h postinfection, the GD strains exhibited faster growth and higher replication titers than the SH strains. The GD23-02 genotype (GD) and SH23-13 genotype (SH) exhibited intriguing amino acid variation in the VP1 E-region. We therefore selected these strains for challenge experiments. Although both strains caused oral-ulcer symptoms, they presented distinct disease progression and clinical manifestations; SH23-13 exhibited traits typical of virulent systemic disease (VSD), whereas GD23-02 predominantly exhibited symptoms of oral respiratory disease (ORD). Notably, both strains consistently induced severe diarrhea and intestinal damage, demonstrating the harmful effects of FCV on intestinal health. GD23-02 exhibited broad-spectrum neutralization capabilities, with antibody titers of 1:168 against the F9 vaccine GI strain and 1:2521 against the SH23-13 GII strain, making this a promising candidate for future vaccine formulation. These findings reveal the genetic diversity and complex pathogenicity of FCV isolates and elucidate the associations between FCV strains and intestinal disease. We recommend incorporating FCV testing into future diagnostic evaluation of feline diarrhea. This testing will be essential in elucidating the tissue specificity of FCV and potential escalation in its virulence.

## 1. Introduction

Feline calicivirus (FCV; *Caliciviridae*) is a highly contagious RNA virus and an excellent model for studying calicivirus biology. FCV is a small, non-enveloped virus (diameter, 30–40 nm), featuring a single-stranded RNA genome of ca. 7.8 kb. The genome is organized into three open reading frames (ORF1, 2, and 3), which encode nonstructural proteins, the major capsid protein VP1, and the minor structural protein VP2, respectively [1]. VP1, the core component of the icosahedral capsid, contains receptor-binding sites and major immunogenic epitopes [2, 3]. E-HVR, a highly variable region (HVR) of VP1 and encoded by amino acids 426–521, is pivotal in the viral infection cycle of FCV; this is because E-HVR facilitates the interaction between VP1 and the cellular receptor-attachment molecule feline junctional adhesion molecule 1 (fJAM-1) [4]. Analysis of VP1 homology and genetic evolution are instrumental in determining the genotype of FCV [5]. FCV exhibits considerable genetic, antigenic, and phenotypic diversity, driven by point mutations, persistent infection, and recombination [6, 7]. Recombination in FCV both contributes to the emergence and evolution of new variants and increases the complexity of clinical symptoms, such as by causing tissue tropism or altering virulence [8, 9].

This diversity complicates the differentiation of pathogenic strains via genetic and serological methods. Two primary FCV phenotypes have been recognized: classical oral respiratory disease (ORD), characterized by symptoms, such as fever, oral ulcers, and conjunctivitis, and virulent systemic disease (VSD), which leads to multi-organ involvement and severe symptoms, such as hemorrhage and lameness [10, 11]. Moreover, recent research has suggested a potential role for FCV in feline intestinal diseases. Tian et al. [12] found that infection with the FCV-2280 strain resulted in intestinal lesions, with infectious particles detectable in the intestinal epithelium, supporting the intestinal tropism of the virus. Based on this evidence, researchers have proposed including FCV in diagnostic algorithms for feline intestinal diseases [13]. Intestinal FCV isolates from cats with enteritis (the E-FCV strain) exhibit higher resistance to extreme in vitro conditions than respiratory isolates (the R-FCV strain) [13, 14]. Cats with panleukopenia and diarrhea exhibited a higher FCV-positivity rate than healthy controls, suggesting that FCV may be a significant component of the feline enteric virome [15]. Nonetheless, the role of FCV as a primary pathogen in intestinal diseases remains unclear.

To address this, we monitored the prevalence of FCV in Shanghai (SH) in 2023 and 2024, identified the molecular characteristics and pathogenicity of the primary FCV isolates, and examined the association between the FCV strains and intestinal disease. These findings, which enrich the molecular epidemiological data on FCV in China, provide powerful evidence to support screening for the VSD- and ORD-related strains of FCV. Further, they provide a theoretical basis for the development of FCV vaccines.

## 2. Materials and Methods

### 2.1. Samples

In 2023 and 2024, we collected samples from 189 domestic cats in SH and from 10 domestic cats in Guangdong (GD), all with upper respiratory tract disease (URTD) symptoms. To identify the respiratory pathogens present, the samples (conjunctival, nasal, and oropharyngeal swabs) were analyzed via reverse transcription quantitative polymerase chain reaction (RT-qPCR) by Zoetis Testing Technology (SH, China). The assays targeted five pathogens—Feline mycoplasma (PPLO), *Chlamydia* (CT), *Bordetella bronchiseptica* (Bp), feline herpesvirus (FHV-1), and FCV.

### 2.2. Virus Isolation and Identification

For samples that tested positive exclusively for FCV, the supernatants were filtered through 0.22 µm filters (MilliporeSigma, Bedford, MA) and inoculated into Crandell–Rees feline kidney (CRFK) cells. The cells were cultured for five passages, with continuous monitoring of cytopathic effects. Upon appearance of cytopathic effects, an indirect immunofluorescence assay was performed using a rabbit polyclonal antibody against the FCV SH-14 VP1 protein. The 50% tissue culture infectious dose (TCID_50_) was determined using the Reed–Muench method. The cells were inoculated with FCV at 100 TCID_50_, and the supernatants were collected at 12, 24, 36, and 48 h postinfection (hpi) for virus-titer determination and growth curve analysis.

To further verify the presence of FCV, 30 mL of the supernatant from the infected cell culture was centrifuged at 6000 × *g* for 30 min to remove cellular debris. The clarified supernatant was subjected to ultracentrifugation at 30,000 × *g* for 120 min to concentrate the virions. The pelleted virions were resuspended and negatively stained with 0.5% phosphotungstic acid for visualization under a Tecnai G2 Spirit BioTwin electron microscope (Thermo Fisher Scientific, Waltham, MA).

### 2.3. Viral Nucleic Acid Extraction and Sequencing

Total viral RNA and DNA were extracted from the culture supernatants using a FastPure Viral DNA/RNA Mini Kit (Vazyme, Nanjing, China). We screened for additional viral pathogens, including FBoV, FeLV, FAstV, FHV-1, FPV, FIV, FRV, FeKoV, FNoV, CPV, CDV, and FCoV, via polymerase chain reaction (PCR). To determine the complete sequence of the FCV isolate, three primer pairs were designed, based on the SH/2014 (KT000003.1) and GXNN02-19 (MZ712022.1) sequences. The primers used in this study are listed in Table 1. Amplification products generated via routine PCR were sequenced by Tsingke Biotech (SH, China).

### 2.4. Sequence Analysis

In total, 136 complete genome FCV sequences were retrieved from the NCBI database and were aligned using MEGA11. Phylogenetic trees were constructed using FastTree, which employs an approximate maximum-likelihood algorithm to deduce evolutionary relationships from nucleotide or amino acid sequence alignments. To assess the reliability of the inferred phylogenetic trees, bootstrap analysis using 1000 replicates was performed, enhancing the statistical robustness of the results. Phylogenetic trees were visualized using the Interactive Tree of Life (iTOL) tool (https://itol.embl.de) [16].

### 2.5. Experimental Determination of Pathogenicity and Assessment of Clinical Signs

Prior to this experiment, the cats were screened for FCV and other viral pathogens via qPCR and were confirmed to be negative. Twelve healthy domestic cats, each 8 months old, were randomly assigned to three groups and each housed in a separate animal facility to mitigate cross contamination risks. To minimize contamination, strict personal hygiene protocols were enforced, requiring the use of single-use protective gear when entering each housing unit. The challenge doses were administered by a senior veterinarian, following the American Animal Hospital Association (AAHA) Anesthesia Guidelines for Dogs and Cats [17]. The cats were anesthetized subcutaneously with a mixture of lidocaine and ketamine at 0.1 mL/kg. For the challenge, each cat was inoculated with 1 × 10^7^ TCID_50_ of either SH23-13 or GD23-02, administered intranasally (0.2 mL per nostril) and ocularly (0.05 mL per eye). The control group was inoculated with an equivalent volume of Dulbecco's modified eagle medium (DMEM) as a mock inoculation.

Pathogenicity was assessed using a scoring system aligned with that described in the European Pharmacopeia to evaluate clinical symptoms, such as oral ulcers, ocular and nasal discharge, body temperature, weight, and mental status (Table 2) [18]. Clinical signs were monitored daily over a 14 days post-challenge period using a scoring sheet, to determine the total and maximum scores for each cat. The aggregate scores were computed by summing the daily scores for the entire observation period. Clinical assessments were conducted independently by two trained veterinarians blinded to the experimental treatments.

### 2.6. Pathogenicity-Experiment Sample Processing and Analysis

Blood samples were collected from the forelimb saphenous vein on days 1, 4, 7, 10, and 13 postinfection, using ethylenediaminetetraacetic acid (EDTA) as an anticoagulant. Complete blood counts and leukocyte differentiation were performed using the ProCyte Dx Hematology Analyzer (IDEXX, Westbrook, ME). Following centrifugation of whole blood at 3000 × *g* for 5 min, the serum was collected and analyzed for serum amyloid A (SAA) using the Vcheck Feline SAA Test Kit (Bionote, Hwaseong, South Korea). Swab samples were collected from the hard palate, tongue, and anorectal region on days 1, 3, 5, 7, 9, 11, and 13 postinfection. Nucleic acids were extracted from swabs and blood samples as described by Luo et al. [19]. Pathological examinations were performed on the lungs and intestinal tissues collected at 7 days postinfection (dpi) from one cat per group and at 14 dpi from two additional cats per group. The tissue samples were fixed, sectioned, and stained with hematoxylin and eosin (H&E) to identify pathological changes attributable to FCV. Intestinal tissue samples obtained from the infected group at 14 dpi were examined via transmission electron microscopy (TEM). At 14 dpi, digital radiography was performed on both the infected and control groups. RT-qPCR was conducted to quantify the viral load, using a CFX Opus 96 Real-Time PCR System (Bio-Rad, Hercules, CA), with the following primers targeting *VP2*: forward, 5′-GCGCAGAAAATTGAATTGGAC-3′ and reverse, 5′-TGTATGAGTAAGGGTCAACCC-3′. At 7 dpi, RT-qPCR data for lung tissue represent technical replicates from a single biological sample per group. At 14 dpi, biological replicates from three animals per group were used for analysis.

### 2.7. Cross-Neutralization Assay

Antigenic relationships among the F9 vaccine strain, SH23-13, and GD23-02 were analyzed via a cross-neutralization assay. Virus-positive sera were mixed with the diluted virus, incubated for 1 h, then added to CRFK cells cultured in a 96-well plate. After incubation for 5 days, cytopathic effects were evaluated and neutralizing antibody titers were calculated.

### 2.8. Statistical Analysis

Statistical analyses were performed using two-way repeated measures ANOVA followed by Tukey's multiple comparisons test in GraphPad Prism 8 (GraphPad Software, La Jolla, CA). Differences were considered significant at *p* < 0.05.

## 3. Results

### 3.1. Molecular Findings and Viral Isolation

**Figure 1:**
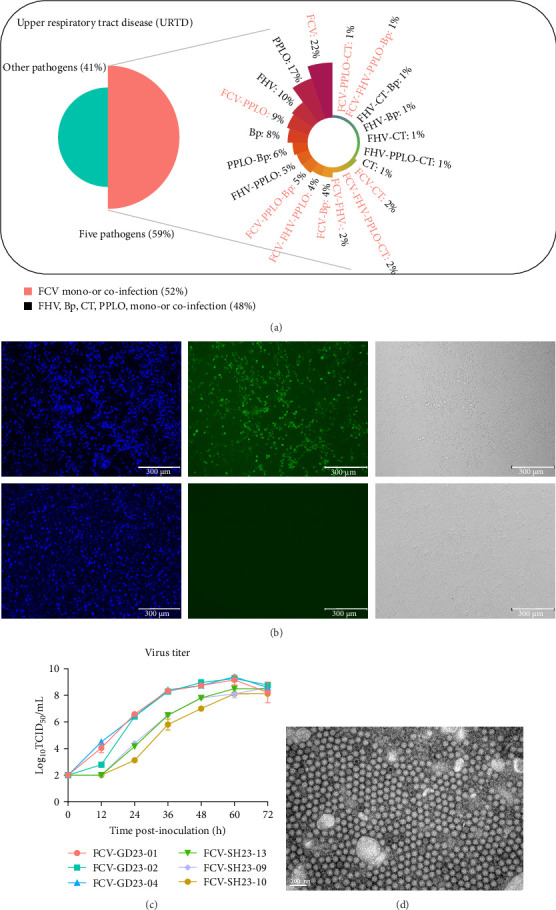
Characterization and growth analysis of feline calicivirus (FCV) isolates. (A) characterization of the common infectious pathogens present in the cats exhibiting upper respiratory tract disease (URTD) symptoms, focusing on FCV positivity alone and in coinfection (*n* = 189). (B) CRFK cells 24 h postinfection with FCV isolates (200× magnification). The presence of green fluorescence, absent in the control CRFK cells, indicates FCV-positivity. (C) dynamic growth curves for the six FCV strains isolated here. (D) transmission electron micrograph of CRFK cells infected with FCV isolates (GD23-02), showing calicivirus virions (diameter, 25–30 nm).

In 2023 and 2024, oral swab samples were collected from 189 cats exhibiting symptoms of URTD, at veterinary clinics in SH, China. The symptoms included stomatitis, conjunctivitis, and in some cases, diarrhea. To identify the cause of the respiratory infection, all samples were sent for respiratory pathogen testing via RT-qPCR; 59% (111/189) of the samples tested positive for the five pathogens tested. Among the positive samples, 52% (58/111) were FCV-positive (Figure 1A). Cats aged 4–6 months were more susceptible to FCV infection, and the incidence among all of the cats sampled was higher during spring and autumn.

We successfully isolated three FCV strains from these samples, designating them SH23-09, SH23-10, and SH23-13. We analyzed these alongside three strains (GD23-01, GD23-02, and GD23-04) isolated from cats with severe respiratory infections in GD in 2023. Inoculation of CRFK cells with these strains produced cytopathic effects within 2–4 days. FCV antigens were detected via an indirect immunofluorescence assay (Figure 1B). In vitro replication kinetics revealed that the viral titers of all six FCV strains peaked 60 h after inoculation, with the isolates from each region exhibiting consistent growth patterns (Figure 1C). During the first 24 hpi, the GD strains exhibited faster growth and higher replication titers than the SH strains. At 24–36 hpi, the SH strains exhibited faster growth than the GD strains; and at 48 hpi, they exhibited similar viral titers. The viral titers of all six strains peaked at 60 hpi, then declined. TEM of the GD23-02 strains confirmed the presence of virions, revealing uniform 30–40 nm particles with dense cores (Figure 1D), thus confirming all six strains as FCV.

### 3.2. Exogenous Virus Detection

The six strains tested positive for FCV but were negative for other common respiratory pathogens, including PPLO, CT, Bp, and FHV-1. Furthermore, additional major feline pathogenic viruses, such as feline panleukopenia virus (FPV), feline immunodeficiency virus (FIV), feline bocavirus (FBoV), feline leukemia virus (FeLV), feline astrovirus (FAstV), feline coronavirus (FCoV), feline rotavirus (FRV), feline kobuvirus (FeKoV), and feline norovirus (FNoV)—as well as canine parvovirus (CPV) and canine distemper virus (CDV), were not detected, ruling out potential interference from exogenous pathogens and confirming the specificity of the FCV isolates.

### 3.3. Phylogenetic Analysis

Using the primers listed in Table 1, we successfully amplified the full genome sequences of the six FCV isolates and submitted the sequences to GenBank (accession numbers OR427299-OR427304).

**Figure 2:**
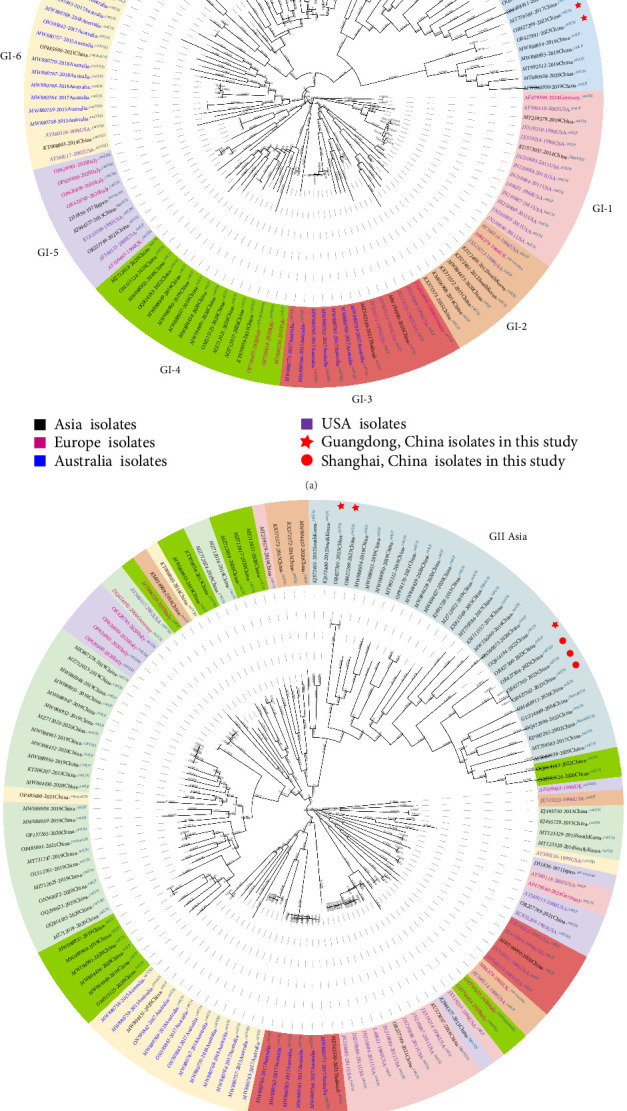
Phylogenetic analysis of the 136 feline calicivirus (FCV) isolates retrieved from the NCBI database. (A) maximum-likelihood (ML) phylogenetic tree based on the capsid VP1 amino-acid sequences of these isolates. (B) ML phylogenetic tree based on the full nucleotide sequences of these isolates. Phylogenetic analysis was performed using FastTree, employing the JTT + G + I model with 1000 bootstrap replicates. Trees were visualized using iTOL. Genotypes are highlighted using color coded backgrounds. Strain origins are distinguished by text color: Asian, black; European, magenta; Australian, blue; U.S., purple. Red symbols, the FCVs isolated here.

To elucidate the evolution of FCV, 138 sequences were analyzed. The *VP1*-based phylogenetic tree clearly divides the FCV strains into two major clades, GI and GII, with our isolates classified as the GII genotype (Figure 2A). Genetic distance analysis (Table 3) revealed significantly greater distances between the GI and GII genotypes (0.184–0.199) than among the GI subtypes (0.165–0.120). This indicates substantial mutations in the GII genotype (which originates from the Asian lineage), leading to the evolution of new strains.

The three SH isolates are closely related, exhibiting a VP1 amino acid genetic distance of 0.0015, whereas a 2018 Australian isolate (MW880769), on the GI-6 branch, is furthest from these three isolates, at 0.2329 (Table S1). The two Guangdong isolates [GD23-04 (OR427301) and GD23-01 (OR427299)] are also closely related (genetic distance, 0.0015), whereas they are substantially further (at 0.2377) from the FCV-255 vaccine strain (U07130), potentially contributing to vaccine immune escape. Interestingly, the genetic distance between the GD isolate GD23-02 (OR427300) and the three SH isolates (0.1113) is smaller than that between the two GD isolates GD23-04 and GD23-01 (0.1811), indicating substantial evolutionary divergence within the same geographic region.

The phylogenetic tree based on FCV whole genome sequence further illustrates the complexity of its evolution, suggesting genomic fragment recombination among different genotypes (Figure 2B). Notably, the recent strains within the Asian lineage form a distinct cluster, highlighting a distinct evolutionary pattern.

### 3.4. Amino Acid Variation in the E-Region of VP1, and Pathogenic Associations

The E-region of VP1, encoded by residues 426–523, is critical for the immunodominance of FCV. Specific residues within this region (438, 440, 448, 452, and 455), located in neutralization sites, may be associated with the differences between the pathogenic types (VSD and ORD) [20]. Sequence analysis revealed that the three SH isolates exhibit four amino acid substitutions (at residues 438V, 440Q, 455T, and 465S), consistent with the VSD phenotype (Table 4). In contrast, the two GD isolates, GD23-04 and GD23-01 exhibit three substitutions (at 438T, 440S, and 465G), typical of the ORD strain.

The GD isolate GD23-02 exhibits a combination of features of the two pathogenic types: residues 438T and 465G are characteristic of the ORD type, whereas residues 440Q and 455T are aligned more closely with the VSD type. This amalgam of amino acid substitutions poses challenges in differentiating between pathogenic strains using genetic methods alone.

### 3.5. Clinical Symptoms and Pathogenicity of GD23-02 and SH23-13

**Figure 3:**
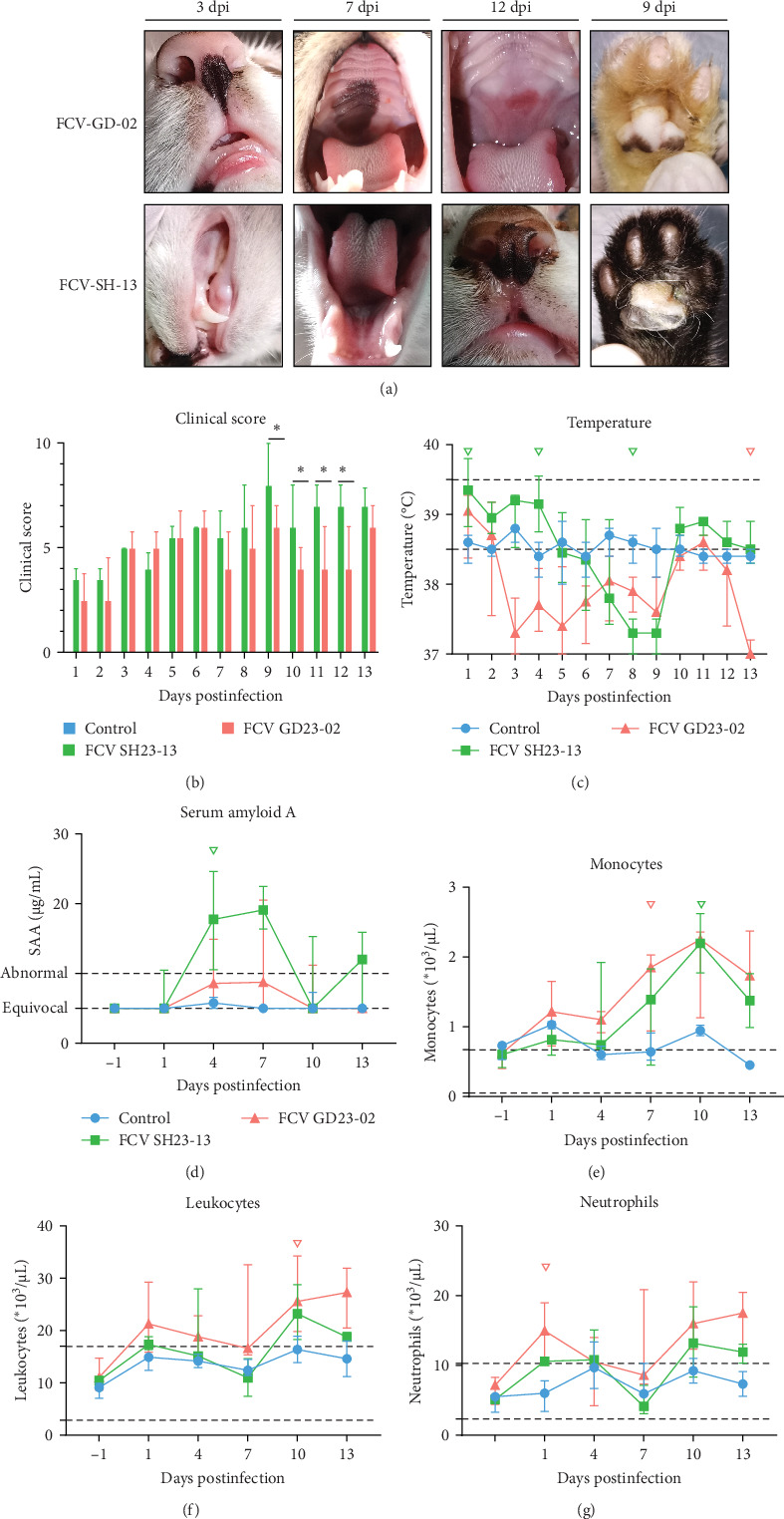
Pathogenicity of feline calicivirus (FCV) isolates SD23-02 and SH23-13. (A) gross lesions observed at various time points postinfection, including oral ulcers, increased nasal secretions, tongue blisters, and swollen footpads. (B) clinical scores and (C) body temperatures in cats (*n* = 4 per group from day 1 to 7; *n* = 3 from day 8 to 13) postinfection. (D) acute-phase serum amyloid A (SAA) levels and (E–G) blood results following FCV challenge. The data are presented as the median ± IQR of the absolute cell counts of leukocytes, total neutrophils (segmented and banded), and monocytes. Whiskers, range from minimum to maximum. The two dotted lines indicate the healthy range. ^▽^*p* ≤ 0.05, infected versus control groups.

We evaluated the pathogenicity of the GD isolate GD23-02 and SH isolate SH23-13. All the infected groups exhibited conjunctivitis, gingival redness, oral ulcers, diarrhea, perianal redness, and inflammation (Figure 3A). The group infected with SH23-13 exhibited distinctive symptoms, including lameness and suppuration of the footpads. Based on clinical scoring, symptoms in the GD23-02 group peaked at 6 dpi, then subsided, exhibiting with periodic fluctuations; in the SH23-13 group, however, they peaked at 9 dpi, then remained stable (Figure 3B). Fluctuations in body temperature were observed from the first day postinfection. In the SH23-13 group, body temperature rose to >39.5°C at 1–4 dpi, declining to <38°C (and stabilizing) by 8–9 dpi (Figure 3C). In the GD23-02 group, body temperature declined consistently, reaching <38°C at 7–9 dpi.

In the GD23-02 group, the white blood cell, neutrophil, and monocyte counts began to increase from 1 dpi, peaking at 10 dpi (Figure 3), with similar patterns in the SH23-13 group. SAA levels were significantly higher in the SH23-13 group than in the GD23-02 group, suggesting that the cats in the two groups experienced different rates of disease progression (Figure 3D).

### 3.6. Lung Damage and Viral Load in GD23-02 and SH23-13 Infection

**Figure 4:**
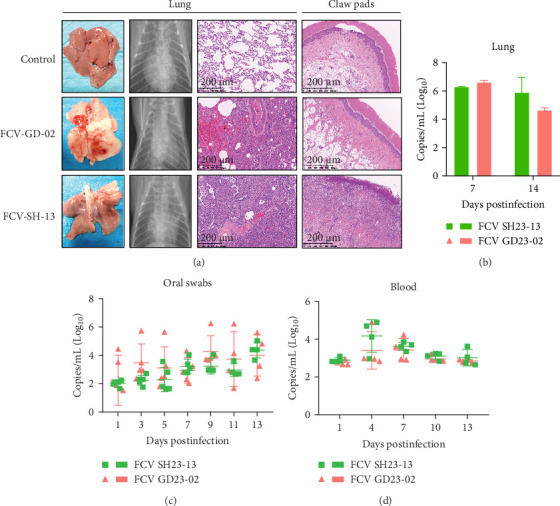
Detection of lesions and feline calicivirus (FCV) RNA in lung, oral swab, and blood specimens. (A) lesion detection via visual examination, digital radiography, and lung histology. (B–D) Viral RNA detection via RT-qPCR following viral challenge, in lung tissue (B), oral swab specimens (C), and blood samples (D). Sample sizes were *n* = 4 per group from days 1 to 7; *n* = 3 per group from days 8 to 13.

Tissue examination at 7 dpi revealed that the GD23-02 isolate caused severe interstitial pneumonia, characterized by extensive inflammatory cell infiltration (Figure 4A), whereas the SH23-13 isolate caused moderate and localized interstitial pneumonia. Tissue staining further revealed epidermal irregularities and extensive inflammatory cell presence in the dermis after SH23-13 infection, symptoms not observed with GD23-02 infection, consistent with the significant footpad suppuration caused by SH23-13. At 7 dpi, the lung viral load was slightly higher in the GD23-02 group than in the SH23-13 group, consistent with the severity of lung damage revealed via histopathology (Figure 4B). However, by 14 dpi, the viral load had decreased in the GD23-02 group, with a corresponding reduction in symptoms.

To track the progression of the infection, nasal swab and blood samples were collected throughout the study period (Figure 4C,D). FCV was detectable in both specimen types at all sampling points. Viral shedding peaked at 9 dpi in the GD23-02 group and 13 dpi in the SH23-13 group. Both groups exhibited peak viremia at 4 dpi.

### 3.7. Intestinal Pathology Induced by GD23-02 and SH23-13

**Figure 5:**
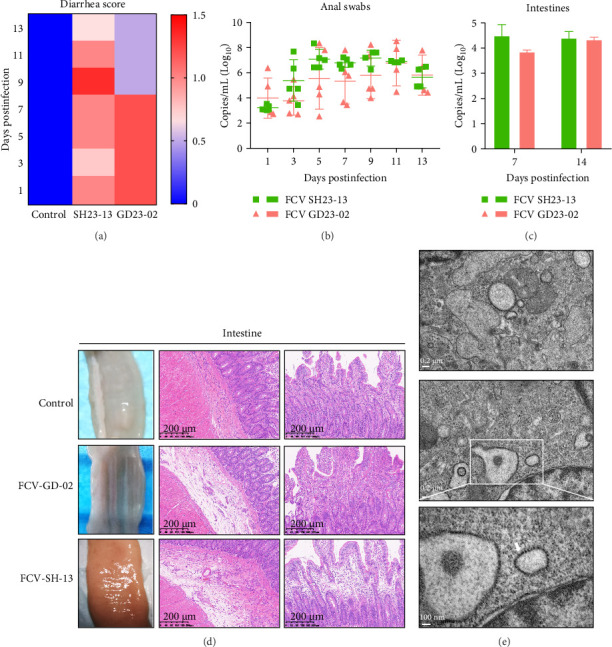
Lesion detection, feline calicivirus (FCV) RNA detection, and ultrastructural analysis of FCV viral particles in intestinal tissue. (A) clinical presentation of diarrhea (*n* = 4 per group from day 1 to 7; *n* = 3 per group from day 8 to 13). Diarrhea severity scores: 1, mild diarrhea and loose stools; 2, severe diarrhea and anal prolapse. (B) viral RNA levels in anal swab specimens. (C) viral RNA levels in intestinal tissue, detected via RT-qPCR, following viral challenge. (D) lesion detection via visual examination and histological analysis of the intestines. (E) examination of intestinal ultrastructure via transmission electron microscopy. The mature viruses are clustered near the cell nucleus (scale bar, 0.2 μm). Area enclosed by the white rectangle (scale bar, 100 nm) reveals the 30 nm viral particles, indicated by white arrows.

Although FCV is traditionally considered a respiratory pathogen, we found that GD23-02 and SH23-13 also caused substantial intestinal damage, causing severe diarrhea (Figure 5A). During the early stage of infection (1–9 dpi), the GD23-02 group exhibited more severe diarrhea than the SH23-13 group, with worse perianal edema and inflammation. In contrast, the SH23-13 group exhibited diarrhea for longer, along with a consistently higher viral load in the intestinal tract (Figure 5B).

FCV was detected in the small intestine in both groups (Figure 5C). In the GD23-02 group, the small intestinal wall was thickened, exhibiting a disordered arrangement of mucosal epithelial cells, indicating tissue damage and regeneration (Figure 5D). In contrast, the SH23-13 group exhibited thinning of the small intestine wall, with bleeding in some places. Histopathological analysis revealed structural damage to the intestinal mucosa, infiltration of lymphocytes and plasma cells, and a significant presence of lymphocytes and macrophages in the connective tissue. TEM of small intestine tissue from the SH23-13-infected group revealed that the nuclei and mitochondria retained their structural integrity (Figure 5E). However, the cytoplasm contained numerous nearly circular, darkly stained particles, ca. 30 nm in size and densely clustered, likely representing FCV particles.

### 3.8. Broad-Spectrum Neutralization Capabilities of GD23-02

**Figure 6:**
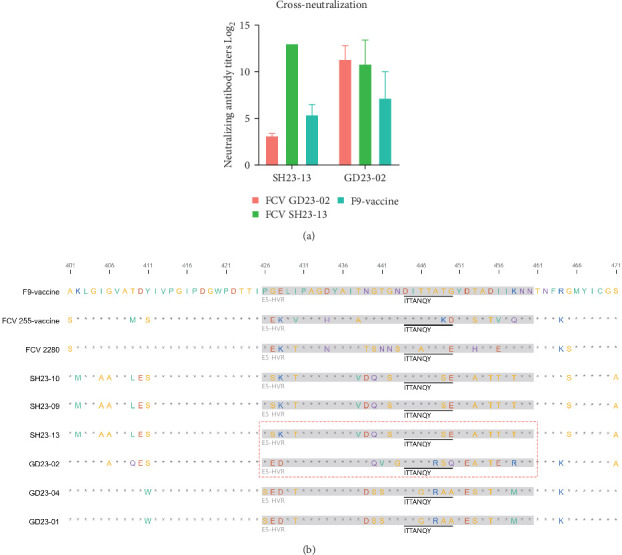
Feline calicivirus (FCV) cross-neutralization assay and sequence alignment. (A) results of cross-neutralization testing among the FCV strains, illustrating the ability of the antibodies to neutralize different strains. (B) amino acid sequence alignment of the capsid sequences of the nine FCV isolates, with the F9 strain sequence for reference. Key epitopes within the sequences, spanning amino acids 426–460 in the E-HVR region and amino acids 445–451 (ITTANQA), in the nine FCV strains, are highlighted.

To assess the potential for serological cross-neutralization between GD23-02 and SH23-13, sera from infected cats were uniformly diluted and tested against each strain. The highest neutralization titers were observed in sera specific to the respective parental strains. Notably, the GD23-02 strain demonstrated broad cross-neutralization capabilities, with titers ranging from 2^7^ to 2^12^ (Figure 6A), exhibiting strong neutralization capabilities against both the SH23-13 and GI F9 vaccine strains. In contrast, SH23-13 strain demonstrated weaker cross-reactivity with the GD23-02 and F9 strains.

## 4. Discussion

FCV, a major pathogen in cats, causes respiratory diseases that can manifest as ORD or VSD. The high genetic variability of FCV, along with the absence of a proofreading function in its RNA polymerase, poses significant challenges in vaccine development. Despite the availability of several FCV vaccines (F9, 21, 255, 431, and G1), the high mutational variability of FCV and its frequent outbreaks in China have cast doubt on the effectiveness of the current vaccines [21, 22].

Here, FCV was detected in 59% of 189 symptomatic cats sampled in SH, confirming the prevalence of FCV infections in the region. Six FCV strains, isolated in 2023 from domestic cats in SH and GD, were phylogenetically analyzed. These isolates were classified within the GII genotype, which appears to be emerging as the dominant genotype in Asia [23]. Notably, the widely used FCV255 vaccine exhibits significant genetic divergence from the isolates identified here (genetic distance, 0.2329), potentially contributing to the reduced vaccine efficacy observed in the field. This is consistent with recent findings suggesting that current GI-genotype vaccines may offer limited protection against the GII genotype prevalent in China [24]. FCV exhibits significant evolutionary adaptability and the potential for cross-species transmission, highlighting the need to monitor genetic variation among FCV strains in order to develop more refined control strategies.

Interestingly, although GD23-02 and SH23-13 exhibit high amino acid sequence homology, they exhibited significantly different neutralization capabilities. This may be due to differences in their antibody levels or to specific differential sites that confer broad-spectrum neutralization against both strains (Figure 6B). Pathogenicity in FCV is associated with E-HVR, within VP1 [11]. We found that the GD isolate GD23-02 is genetically closer to the SH isolate SH23-13, with which it shares four amino acids (440Q, 453A, 455T, and 492L) in the critical E-HVR region, than to the other GD isolates. However, GD23-02 and SH23-13 function as distinct pathogens in cats. Although both cause severe oral ulcers and respiratory symptoms, GD23-02 exhibits a more acute onset, distinctly alters routine blood levels, and tends to cause chronic stomatitis, whereas SH23-13 exhibits a slower onset, with severe symptoms (including footpad blisters and lameness) developing later.

FCV has previously been classified into respiratory and intestinal types, based on its isolation site and bile acid resistance [13, 14]. Here, however, the presence of FCV RNA in oral and anal swab and blood specimens from throughout the infection period, and particularly in those isolates initially identified from oral swabs, challenges the validity of this classification. It has recently been revealed that FCV is a frequent component of the feline enteric virome, occurring in 25.9% of diarrheal feces, commonly coinfecting with gastroenteritis pathogens, such as FPV, FCoV, and FAstV [15]. Although FCV is well-known as a respiratory pathogen, its specific enteric pathogenicity remains underexplored, with few studies or infection-model analyses linking gastrointestinal symptoms to FCV [25, 26].

Based on our findings, the GD23-02 and SH23-13 FCV strains examined here can cause severe diarrhea and mild rectal prolapse, accompanied by bleeding spots and changes in the thickness of the small intestine wall. H&E staining and TEM revealed substantial macrophage infiltration and viral particle aggregation in intestinal tissue, highlighting the detrimental effects of the virus on intestinal health. While previous reports of enteric FCV isolates, mostly from the feces of symptomatic cats, have lacked details on pathogenicity, our findings are consistent with findings for the FCV-2280 strain, which is known to induce intestinal lesions [12]. We recommend incorporating FCV testing into the diagnostic evaluation of feline diarrhea. Further research is required to elucidate the enteric pathogenicity of FCV. In particular, molecular markers that distinguish between the enteric (E-FCV) and respiratory (R-FCV) isolates should be identified. This is essential to elucidate the tissue specificity of FCV and potential escalation in its virulence.

Differences among viral strains in adaptability, mutation rates, immune escape strategies, and surface specificity lead to poor cross-neutralization and vaccine protection. Given the broad-spectrum neutralizing capability of GD23-02, especially against prevalent GII genotype strains and established vaccine strains (e.g., FCV-F9), integrating GD23-02 into a multivalent vaccine strategy is promising. Such formulations, combining GD23-02 with antigenically complementary strains like SH23-13, could improve vaccine-induced cross-protection [6]. Additionally, GD23-02 offers an optimal genetic backbone for reverse genetics approaches, facilitating the targeted engineering of attenuated or epitope-optimized strains. The highly variable 5′ and 3′ regions of the FCV capsid protein gene exhibit immune-mediated selection involving B cells [4]. The amino acid 445–451 region in VP1 is particularly important for neutralization, exhibiting high sequence diversity under strong immunization-selection pressure. Further research is required to clarify whether GD23-02 and SH23-13 share identical or similar antigenic epitopes in this region, leading to an effective immune response, and whether they exhibit common mutation strategies in multiple immune-escape-related regions.

## 5. Conclusions

Six FCV strains, isolated from domestic cats in SH and GD in 2023, exhibited the GII genotype, suggesting that this genotype is becoming predominant in Asia. Pathogenicity testing identified GD23-02 as the ORD pathotype and SH23-13 as the VSD pathotype, which is marked by lameness. Despite the distinct differences in disease progression and clinical manifestations between these strains, both strains consistently induced severe diarrhea, thereby linking FCV to intestinal pathology and potentially revealing the presence of a third, intestinal-specific pathotype. Research into the unique molecular epidemiological traits of these Chinese FCV variants is crucial for disease management and to elucidate the evolutionary dynamics of caliciviruses.

## Figures and Tables

**Table 1:** Primers used in this study.

Primer	Sequence (5′–3′)	Size of PCR product (bp)
FCV-ORF1-F1	TTGAGACAATGTCTCAAACT	2472
FCV-ORF1-R1	GAAGAGCCCAGGCCAAATCAAA	
FCV-ORF1-F2	CTACCCGCCAATCAACATGTGGTA	2920
FCV-ORF1-R2	AGCACGTTAGCGCAGGTTGA	
FCV-ORF23-F1	ACTGTGATGTGTTCGAAGTTTGAG	2393
FCV-ORF23-R1	CCCTGGAGTTAGGCGCAAATG	
qFCV-VP2F	GCGCAGAAAATTGAATTGGAC	199
qFCV-VP2R	TGTATGAGTAAGGGTCAACCC	
FHV-1-F	TCCAAAATTCTATGCGGGTGA	515
FHV-1-R	TAAGACCACCTTGCCGA	
FPV-F	TACATGGCAAACAAATAGAGC	694
FPV-R	ATTTGTTAAATTAGGCGCAAC	
FBoV-F	AGAACCRCCRATCACARTCCACT	465
FBoV-R	TGGCRACCGCYAGCATTTCA	
FeLV-F	TGAATGTCCCTACCCGTT	601
FeLV-R	GGATGGATGTCCTCTACCC	
FCV-F	AACCTGCGCTAACGTGCTTA	924
FCV-R	CAGTGACAATACACCCAGAAG	
FAstV-F	GCGGATTGGGCATGGTTTAGA	645
FAstV-R	ACCCCTCGTTTGGATCGTTACCT	
FCoV-F	TGTCAACGCGACTGTAATTG	389
FCoV-R	CAACAACTTCCTAAACAACC	
FNoV-F	GATTACTCCASSTGGGAYTCMAC	319
FNoV-R	TGACGATTCATTCATCMCCRTA	
FeKoV-F	CATGCTCCTCGGTGTTCTCA	611
FeKoV-R	GTCCGGGTCCATCACAGGGT	
CPV-F	GGATGGGTGGAAATCACAGC	845
CPV-R	ATAACCAACCTCAGCTGGTC	
FRV-F	TGGCCAGCGCCAGTGACAAA	421
FRV-R	TACTGGGTTGTTGTTTAACGT	
CDV-F	CGAGTCTTTGAGATAGGGTT	455
CDV-R	CCTCCAAAGGGTTCCCATGA	
FIV-F	AATGTAWCTACAGGACGAGAA	433
FIV-R	TCCTARYCCWTCTCTWGCYTTYTCCA	

**Table 2:** Clinical score sheet.

Clinical sign	Severity of symptoms	Score
Oral ulcer	Severity (large, ≥4 mm diameter)	2
	Slight (single or multiple, <4 mm diameter)	1
	No	0

Eye and nose discharge	Severity	2
	Slight	1
	No	0

Body temperature	>40°C or <38°C	2
	38–38.5°C or 39.5–40°C	1
	38.5–39.5°C	0

Body weight	Reduction of 10% and above	2
	Within 10% reduction	1
	Steady increase	0

Mental state	Mental depression and loss of appetite	2
	Mental depression or loss of appetite	1
	Normal	0

Diarrhea	Severity	2
	Slight	1
	No	0

Other symptoms	Severity	2
	Slight	1
	No	0

**Table 3:** Evolutionary divergence metrics for genotype pairs.

Genotype	GI-1	GI-2	GI-3	GI-4	GI-5	GI-6	GI-7	GII
GI-1	—	—	—	—	—	—	—	—
GI-2	0.124	—	—	—	—	—	—	—
GI-3	0.120	0.137	—	—	—	—	—	—
GI-4	0.130	0.137	0.149	—	—	—	—	—
GI-5	0.132	0.141	0.135	0.149	—	—	—	—
GI-6	0.133	0.149	0.141	0.144	0.149	—	—	—
GI-7	0.151	0.162	0.146	0.165	0.159	0.154	—	—
GII	0.184	0.196	0.188	0.194	0.191	0.199	0.196	—

**Table 4:** E-region capsid protein VP1: hypothesized orchestrator of FCV pathotype shift from oral respiratory disease (ORD) to virulent systemic disease (VSD).

Strains	Amino acid position on VP1 associated with VSD pathotype						
	438	440	448	452	455	465	492
VSD	**V**_13_T_29_I_2_	**Q**_7_G_18_E_11_S_4_T	**K**_15_ **R**_6_A_10_G_2_P_10_E	**E**_22_D_22_	**T**_8_D_19_K_6_M_2_I_4_A_2_NES	**S**_31_G_13_	**V**_31_L_4_I_7_KR
ORD	**T**_46_V_4_A	**G**_23_S_12_Q_3_R_4_D_4_E_2_N_2_	**A**_38_P_4_G_2_K_2_R_5_	**D**_44_E_7_	**D**_32_T_8_S_3_G_3_NEIAV	**G**_40_S_11_	**V**_31_L_8_I_2_R_6_K_3_A
SH23-09	**V**	**Q**	A	D	**T**	**S**	L
SH23-10	**V**	**Q**	A	D	**T**	**S**	L
SH23-13	**V**	**Q**	A	D	**T**	**S**	L
GD23-02	T	**Q**	R	**E**	**T**	G	L
GD23-01	T	S	R	**E**	**T**	G	**V**
GD23-04	T	S	R	**E**	**T**	G	**V**

*Note*: Residues in bold indicate correspondence with the VSD pathotype configuration. Subscript numbers indicate the frequencies from the original alignment [20].

## Data Availability

The data that support the findings of this study are openly available in GenBank at https://www.ncbi.nlm.nih.gov/nuccore/OR427304.1/ (accession number: OR427304.1).

## Supporting Information

Supporting Information Table S1 presents the genetic distance analysis of capsid (VP1) gene amino acid sequences.
